# Choline Acetate/Water Mixtures: Physicochemical Properties and Structural Organization

**DOI:** 10.3390/molecules30163403

**Published:** 2025-08-18

**Authors:** Emanuela Mangiacapre, Zina Barhoumi, Martin Brehm, Franca Castiglione, Valerio Di Lisio, Alessandro Triolo, Olga Russina

**Affiliations:** 1 Chemistry Department, University of Rome La Sapienza, 00185 Rome, Italy; emanuela.mangiacapre@uniroma1.it; 2 Department of Chemistry, University of Tunis El Manar, Rommana 1068, Tunisia; barhoumizina46@yahoo.com; 3 Chemistry Department, Paderborn University, Warburger Strasse 100, 33098 Paderborn, Germany; martin_brehm@gmx.de; 4 Department of Chemistry, Materials and Chemical Engineering “Giulio Natta”, Polytechnic University of Milan, 20133 Milan, Italy; franca.castiglione@polimi.it; 5 Donostia International Physics Center, Paseo Manuel de Lardizabal 4, 20018 San Sebastián, Spain; valerio.dilisio@dipc.org; 6 Istituto Struttura della Materia—Consiglio Nazionale delle Ricerche (ISM-CNR), 00133 Rome, Italy

**Keywords:** DESs, green solvents, X-ray scattering, AIMD, hydrogen bonding

## Abstract

In the quest for greener alternatives to conventional organic solvents, Deep Eutectic Solvents (DESs) have gained significant attention due to their sustainability, biodegradability, and tunability. The use of water as an active and genuine component has recently led to the emergence of water-based DESs (wb-DESs). Here, a careful experimental characterization was performed on choline acetate (ChAc)/water mixtures across a range of water:ChAc molar ratios (n = 2–6). Differential Scanning Calorimetry (DSC) revealed glass transitions between 150 and 180 K, with no first-order transitions, leading to a classification of these mixtures as Low Transition-Temperature Mixtures (LTTMs). Physicochemical measurements, including density, viscosity, electrical conductivity, and refractive index, were conducted over a broad temperature range. NMR analyses provided insights into dynamics and solvation environments, with ^1^H T1_slow_ relaxation times reaching their lowest value at n = 2, consistent with the formation of a strong hydrogen-bonding network. The n = 2 mixture was further investigated using Small and Wide-Angle X-ray Scattering (S-WAXS) and ab initio molecular dynamics (AIMD). These studies, jointly with ^1^H NMR choline diffusion coefficient, directly challenge previous claims of the existence of aggregation phenomena in wb-DES. The simulation revealed a well-organized solvation structure, where acetate and water synergistically stabilize the choline cation through a cooperative hydrogen-bonding network. These findings highlight the impact and significance of an integrated physicochemical study in guiding the rational development of new sustainable systems, such as wb-DESs.

## 1. Introduction

In recent years, the development of alternative solvent systems has gained increasing attention due to the sustainability issues associated with conventional organic solvents. In this context, Deep Eutectic Solvents (DESs) have emerged as innovative and “green” alternatives for a wide range of applications [1,2]. The term “DES” was introduced in the early 2000s by Abbott et al., who reported a eutectic mixture of choline chloride and urea with a melting point significantly lower than those of the individual components [3].

DESs are now recognized as a subclass of eutectic solvents, characterized by mixtures of two or more components forming a liquid with a melting point well below that predicted by ideal eutectic behavior [4,5]. This depression is attributed to strong inter-species interactions, typically hydrogen bonds, between a hydrogen bond donor (HBD) and a hydrogen bond acceptor (HBA) [6]. The appeal of DESs lies in their low cost, easy preparation, and tunable properties, which can be tailored by selecting appropriate HBDs and HBAs. Their design flexibility, combined with features such as low toxicity, non-flammability, and biocompatibility, further enhances their attractiveness for sustainable applications [7]. DESs can be classified into five types based on the nature of the HBA and HBD components. Types I, II, and III all feature a quaternary ammonium salt as the HBA, differing only in the HBD: Type I uses metal chlorides, Type II uses hydrated metal chlorides, and Type III employs small organic molecules. Type IV combines features of Types II and III, with hydrated metal chlorides as HBAs and small organic HBDs. Type V consists entirely of non-ionic species acting as both HBA and HBD [6,8].

The most common type is Type III, where choline chloride (ChCl) reigns supreme as the quaternary ammonium salt used [3,9,10,11,12,13,14]. ChCl is widely used as a nutritional additive and is considered safe for both human and animal consumption. It is classified as a GRAS (Generally Recognized as Safe) substance by the FDA (Food and Drug Administration) and is approved for use in food and animal feeds. Accordingly, ChCl-based DESs are generally considered non-toxic and biodegradable [15]. The combination of ChCl and water has recently led to the development of a new class of sustainable deep eutectic mixtures, known as water-based DESs (wb-DESs) [16,17]. In parallel, the use of alternative anions with choline has been explored. Notably, choline acetate (ChAc), a fully bio-derived ionic liquid (IL), has been proposed as a biocompatible and cost-effective DES component [18]. ChAc was efficiently utilized as a HBA in conjunction with several HBDs, including urea, ethylene glycol, and glycerol, to create DESs [19,20], particularly for enzyme stabilization and increase in their activity [21,22,23,24,25], but also in the framework of hemicellulose solubilization [26]. Recently, due to the strong water affinity of ChAc, its combination with water was utilized for the extraction of several algicidal chemicals from mango waste [27]. Additionally, a combined thermophysical analysis and DFT (Density Functional Theory) calculations were carried out to investigate solute–solvent interactions in highly diluted aqueous solutions of ChAc, revealing the presence of strong ion–water interactions across a wide concentration range [28]. The combination of water and ChAc has been investigated in electrochemistry, particularly for developing aqueous electrolytes. Studies have shown that ChAc–water mixtures perform well in Zn–ion devices, achieving capacities around 200 mW·h·g^−1^ over 20 cycles [29], and that a 30 wt% ChAc solution functions effectively as an electrolyte in Zn–air cells, showing good reversibility and energy efficiency [30]. Moreover, ion–ion and solvent interactions of ChAc in both water and DMSO have been studied via concentration-dependent NMR, to support its application in Zn–air battery technologies [31]. The mixture of ChAc and water was also studied in the context of DESs formation. Miao et al. investigated numerous ChAc aqueous solutions with varied percentages of water and identified the ChAc/water mixture with the 35 wt% of water as the one having the deep eutectic behavior [32].

Given the necessity of developing new sustainable and environmentally appropriate DESs like the aforementioned wb-DESs [16,17,33], this study presents a thorough physicochemical analysis of various ChAc/water mixtures with diverse water:ChAc molar ratios (n) in a range between 2 and 6. First, a thermodynamic analysis was carried out using Differential Scanning Calorimetry (DSC) measurements, comparing with the corresponding findings from the Miao et al. data set [32]. Next, density, viscosity, electrical conductivity, and refractive index of all the mixtures were measured between 278 and 323 K. Finally, ^1^H NMR experiments were utilized to calculate chemical shifts, relaxation times, and the choline, acetate, and water diffusion coefficients as a function of water content. Despite the lack of experimental data for systems with n < 2, NMR experiments suggested a more rigid hydrogen-bonding network for n = 2, likely indicative of DES-like behavior. On this basis, the structural organization of the n = 2 mixture was investigated through a combined approach involving X-ray scattering experiments (SAXS and WAXS) and ab initio molecular dynamics (AIMD) simulations.

## 2. Results

**Figure 1:**
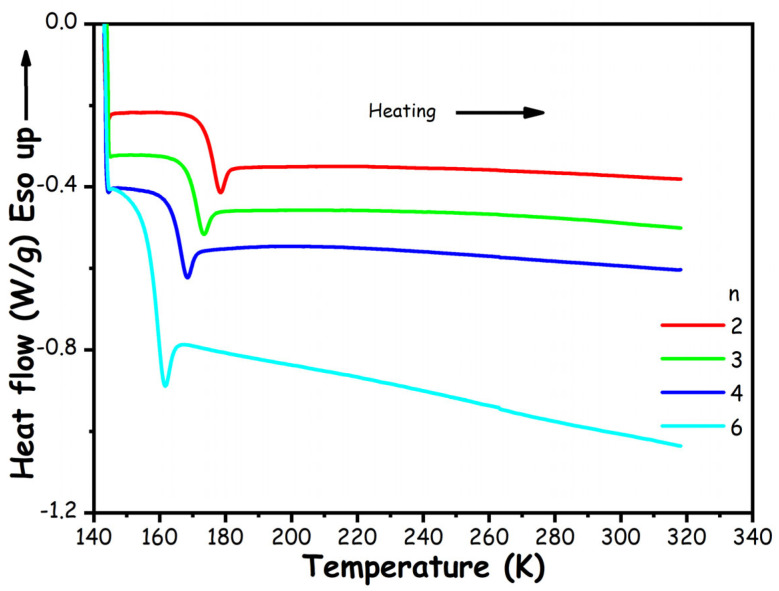
DSC heating (10 K/min) traces of ChAc/water mixtures at different molar ratios n.

The thermal behavior of ChAc/water mixtures at different molar ratios n (n = 2–6) has been monitored by Differential Scanning Calorimetry (DSC) measurements. Heating traces are shown in Figure 1.

Figure 1 shows that the thermograms consistently reveal merely the presence of a glass transition (T_g_), in the range 150–180 K. This second-order phenomenon occurs when temperature rises and an amorphous solid transforms into a subcooled liquid. In particular, in the present data sets, the glass transition is superimposed to an enthalpic recovery endotherm, which manifests as an endothermic peak. This feature is typically due to sample aging. The present experiment set did not allow detection of the crystallization events and/or the corresponding melting ones: the mixtures invariably maintained amorphous/liquid within the investigated temperature range, far below the melting temperatures of both ChAc (362.63 K) and water (273.15 K). This allows us to reliably identify our systems at least as Low Transition-Temperature Mixtures (LTTMs), as it is not possible to discern solid–liquid equilibria data that might prompt for (deep) eutectic behavior. At the present stage, we can speculate that highly concentrated salt mixtures are characterized by a rigid environment, associated to strong ion–ion correlations, only marginally separated by water, leading to high glass transition temperature. Upon water addition, a less constrained and rigid environment develops, where water progressively develops a bulk-like hydrogen bonding network and breaks apart the ionic network constraining the ionic species, thus leading to a decrease in glass transition, hence, a more fluid system.

In this context, we can compare (Table S2 and Figure S1 in the ESI) our results for T_g_, with Miao et al. thermal transitions reported for ChAc/water mixtures [32]. Therein, we show the values obtained for the glass transition, T_g_ (which follow a rather linear dependence from n) and those associated to the transition indicated as melting by Miao et al. [32], T_tr_. Those authors obtained their transition temperatures as “maxima of the melting endotherms”. It emerges that a reasonable agreement exists between their T_tr_ and our T_g_ values, thus suggesting that rather than melting points, those transitions might refer to enthalpy recovery features embedded into the glass transition. If this is the case (no DSC traces were provided in reference [32]), no indication that ChAc/water mixtures might be characterized by solid–liquid equilibria has been obtained in the literature and only glass transitions can be detected. Hence, the previous claim that ChAc/water mixture with n = 5 corresponds to a DES is not substantiated.

From a thermodynamic perspective, our results can be compared with those from other ChAc-based mixtures. When ChAc is mixed with glycerol, ethylene glycol, and urea at different molar ratios it shows not only distinct glass transitions but also melting points [21]. On the other hand, our observations align with the results obtained for the ChAc:U (1:2) system, which displays a liquid–glass transition temperature of approximately 223 K upon cooling [20]. Additionally, our results are consistent with findings from various ChAc-based LTTMs, containing different hydrogen bond donors—including urea, glycerol, and ethylene glycol (1:2 ratio)—where either glass transition or no transition at all, down to 190 K, have been reported [22].

**Figure 2:**
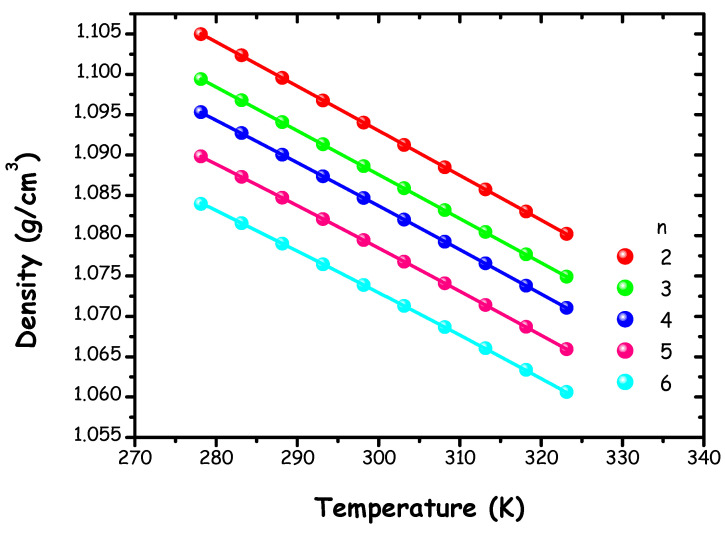
Density (ρ) values of ChAc/water mixtures at different molar ratios n as function of temperature. The solid lines represent the description of experimental data in terms of the Equation (1).

Figure 2 presents the density (ρ) values (Table S3 of the ESI) for the tested ChAc/water mixtures (n = 2–6) as a function of temperature in the range 278.15 K to 323.15 K.

The relationship between density and temperature was evaluated through a quadratic relationship, described as follows:

(1) $$\rho \left(T\right)=\rho_{0}+BT+AT^{2}$$

where ρ (g/cm^3^) indicates the experimental density of the mixtures, while ρ_0_, B and A represent fitting parameters (Table S4 of the ESI).

A linear model is commonly used to describe the relationship between density and temperature in various DESs and/or LTTMs containing different choline salts, particularly choline chloride [13,34,35,36]. Our study opts for a quadratic model in the ρ(T) trend. This choice has been shown to result in more accurate modelling, especially across a broad temperature spectrum, similarly to the approach used in reference [16]. For comparison, Polomski et al. and Hoppe et al. have reported density measurements at 298.15 K for various ChAc-systems using diverse HBD species at 1:2 molar ratio and obtained higher density values than our study, consistent with the different nature of the involved HBD species [19,22].

Density values for each mixture at 298 K as a function of the molar ratio n are reported in Figure S2 of the ESI. A linear trend is observed between density and n, in agreement with our previous findings on ChCl/water systems within the same concentration range [16].

We also mention related density data reported by Veroutis et al., who conducted a physicochemical characterization of diluted aqueous (D_2_O) solutions of ChAc. Specifically, they measured the density of various ChAc and D_2_O mixtures across a range of salt concentrations, from 6 mM to 796 mM (corresponding to the n range between 70 and 9000). Their study reported an essentially constant density value of ρ = 1.1058 g/cm^3^ at ca. 296 K [31].

The thermal expansion experienced by the explored mixtures due to temperature increase can be described by the volumetric thermal expansion coefficient α. This coefficient is defined at constant pressure as:

(2) $$\alpha_{P}=-\frac{1}{\rho (T)}\cdot {\left(\frac{\delta \rho (T)}{\delta T}\right)}_{P}$$

Based on the density–temperature relationship outlined in Equation (1), the α coefficient can be determined as follows:

(3) $$\alpha_{P}=\frac{-(2AT+B)}{AT^{2}+BT+\rho_{0}}$$

Figure S3 in the ESI shows the thermal expansion coefficients as a function of temperature for each mixture. As illustrated, the thermal expansion coefficient displays a clear monotonic trend with increasing temperature, analogously to the ChCl/water system [16]. The rate of change, and thus the slope, increases as the water content increases, ultimately approaching the behavior of pure water. Results in Figure S3 suggest that an isosbestic point exists at ca. 320 K. This observation is comparable to the ChCl/water case, where the curves intersected at ca. 315 K [16], suggesting a thermally activated onset of local and transient tetrabonded hydrogen bond networks in water at lower temperatures [37]. However, for the ChAc/water mixtures, this point is less clearly defined, likely due to the more limited range of mixtures explored in this study.

**Figure 3:**
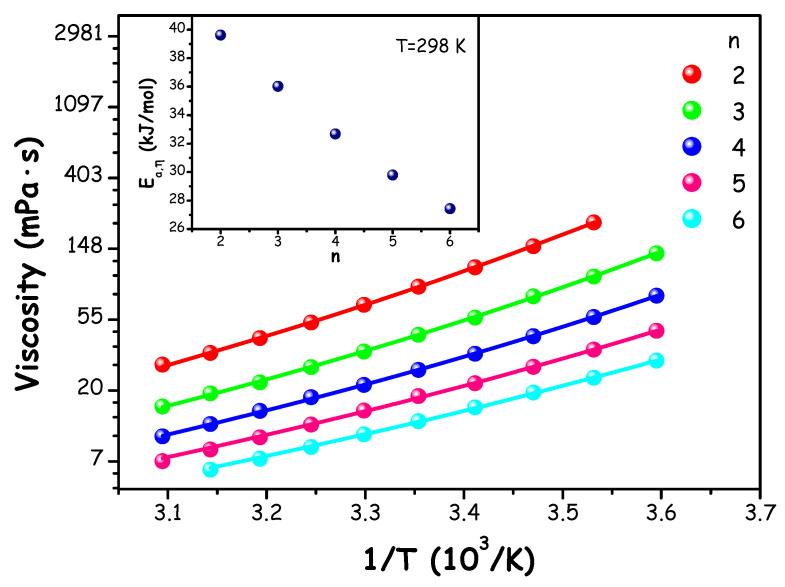
Dynamic viscosity (η) of ChAc/water mixtures at different molar ratios n as a function of temperature. The solid lines represent the description of experimental data in terms of the Equation (4). In the inset, the energy of activation for viscous flow as a function of molar ratios n at 298.15 K.

Dynamic viscosity (η) experiments were performed on the ChAc/water mixtures (n = 2–6) over a temperature range of 278.15 K to 323.15 K (Table S5 of the ESI). Figure 3 displays the resulting data, which were modelled using the Vogel–Fulcher–Tamann (VFT) model:

(4) $$ln\eta =ln\eta_{0}+\frac{D}{T-T_{0}}$$

In (4) η_0_, D and T_0_ are the fitting parameters, representing the viscosity value at the high-temperature limit, a quantity associated to the activation energy, and the so-called “Vogel temperature”, respectively. Fitting parameters are reported in Table S6 of the ESI.

Dynamic viscosity is one of the fundamental properties of (deep) eutectic solvents, since it plays a key role in developing solvents’ applications; it is noteworthy that ChAc/water mixtures possess substantially lower dynamic viscosity values than other ChAc-based eutectic mixtures [19,22].

To have a better understanding about dynamic viscosity, the activation energy for viscous flow, E_a,η_, at T = 298.15 K was estimated, using the relationship:

(5) $$E_{a,\eta }=R\frac{dln\eta }{d(\frac{1}{T})}=RD({\frac{T}{T-T_{0}})}^{2}$$

where R denotes the gas constant (R = 8.3145 J·mol^−1^·K^−1^).

Assuming viscosity is a thermally driven process, E_a,η_ is a physical magnitude indicating how quickly viscosity changes with temperature. Thus, this value represents the energy activation barrier that a species must overcome when exposed to a viscous flow [16,38]. In the inset of Figure 3, this energy barrier is displayed as a function of n at a temperature of 298.15 K, revealing a clear linear decrease as water content grows.

Finally, in Figure S4 of the ESI, dynamic viscosity values as function of n are reported. As expected, we notice a decrease in dynamic viscosity as n increases [16]. We mention that Veroutis et al. observed significantly lower dynamic viscosity values for their highly dilute D_2_O solutions of ChAc at 23.5 °C [31].

**Figure 4:**
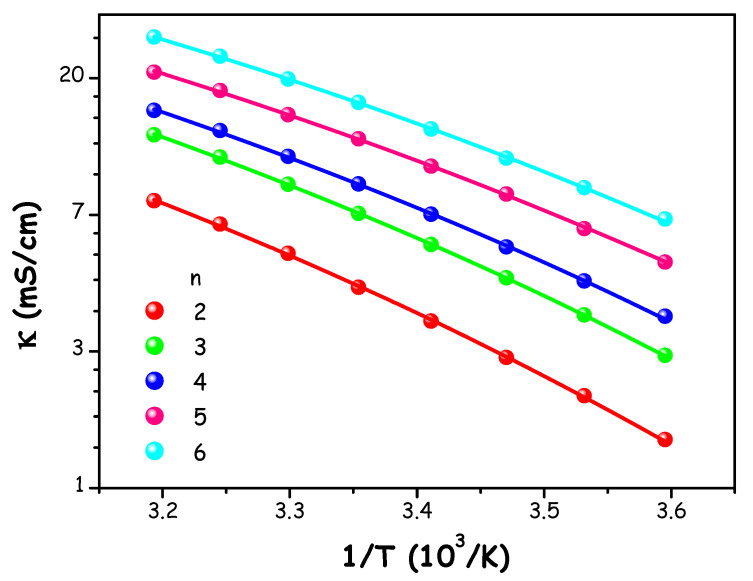
Electrical conductivity values (κ) of ChAc/water mixtures at different molar ratios n as a function of temperature. The solid lines represent the description of experimental data in terms of the Equation (6).

The electrical conductivity (κ) of ChAc/W mixtures (n = 2–6) was assessed within the temperature range of 278.15 K to 313.15 K (Figure 4), with corresponding values provided in Table S7 of the ESI.

Given the relationship between conductivity and dynamic viscosity, we modelled the conductivity temperature dependence by the VFT model:

ln κ = ln κ_o_ + D_κ_/(T − T_κ_) (6)

here, κ_o_, T_κ_, and D_κ_ represent fitting parameters as listed in Table S8 of the ESI.

When comparing our findings with those reported by Polomski et al. and Hoppe et al., who investigated numerous binaries and ternary ChAc-based systems featuring various types of HBDs, we observe higher conductivity values at 298.15 K. Specifically, the first study reported a conductivity range of 0.24–9.93 mS/cm, whereas the second study showed a range of 0.34–4.60 mS/cm [19,22].

Finally, these mixtures were analyzed to investigate the relationship between electrical conductivity and dynamic viscosity. Specifically, molar conductivity (Λ = κ·M/ρ, where M is the molar mass of the mixture and ρ is the density) can be related to fluidity, η^−1^ through the Walden product Λ∙η^α^ = const (data are reported in Table S9 of the ESI). Here, α denotes the decoupling constant, while “const” is a temperature-dependent constant. When α approaches 1, it indicates that molar conductivity scales proportionally with fluidity, reflecting efficient ion mobility and, consequently, good ionicity. To determine the α value for each ChAc/water mixture, a linear fit of log(Λ) versus log(η^−1^) was performed considering for each n a temperature range of 278.15 K–313.15 K. Specifically, the following linear relation was considered:

(7) $$\log\left(\Lambda \right)=\alpha \log\left(\eta^{-1}\right)+const$$

The resulting α values for each investigated ChAc/water mixture are presented in Table S10 of the ESI. As shown in Table S10, the α values for all investigated ChAc/water mixtures approach unity, thus suggesting that these systems exhibit good ionic behavior. This indicates that ion diffusion occurs relatively freely, resembling the behavior observed in conventional aqueous salt solutions.

**Figure 5:**
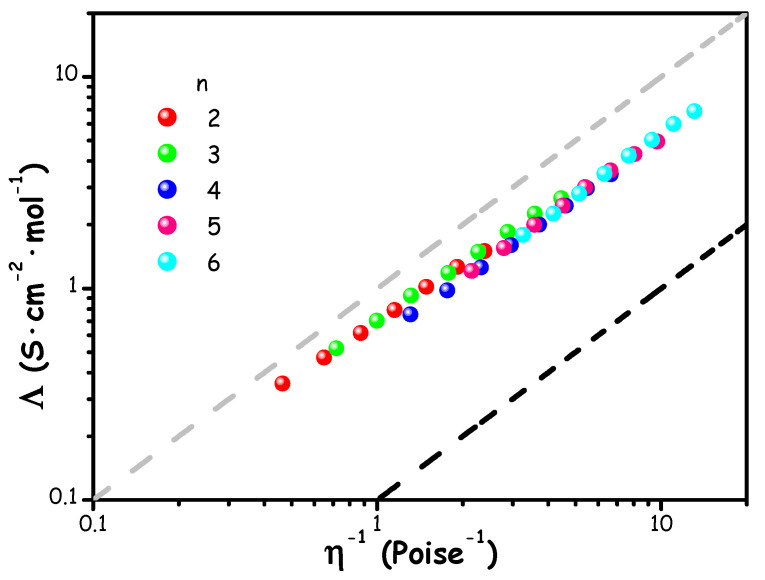
Walden plot of ChAc/water mixtures at different molar ratios n and in a temperature range between 278.15 and 313.15 K. The grey dashed line represents the ideal trend for a 1 M aqueous KCl solution, the black line represents a vertically shifted (by an order of magnitude) ideal trend.

The log(Λ) vs. log(η^−1^) representation is, by definition, the Walden plot (Figure 5).

This type of representation is commonly used in electrochemistry, particularly for organic electrolytes containing lithium salts [39,40,41], ionic liquids (ILs) [42,43,44,45], and more recently in the context of (deep) eutectic mixtures [46,47,48,49]. It allows the graphical evaluation of ionic dissociation in a system by comparison with an ideal reference line, typically derived from dilute aqueous KCl solutions, where ionic interactions are assumed to be negligible. The grey line in Figure 5 represents the “1 M KCl reference”, which corresponds to fully dissociated ions (α = 1), while the black line, parallel to the first but displaced downward by one order of magnitude, suggests that 90% of the ions are paired. Mixtures located between these two lines are considered to have “good ionicity”, highlighting the large population of unpaired ionic species in the mixture [50,51].

The trend observed in Figure 5 indicates that, across the probed water content levels, the mixtures exhibit remarkable ionic behavior, with only slight deviations from the KCl reference trend. Thus, in all the mixtures, ions present high mobility, thereby supporting the assumption confirmed by the results reported in Table S10.

**Figure 6:**
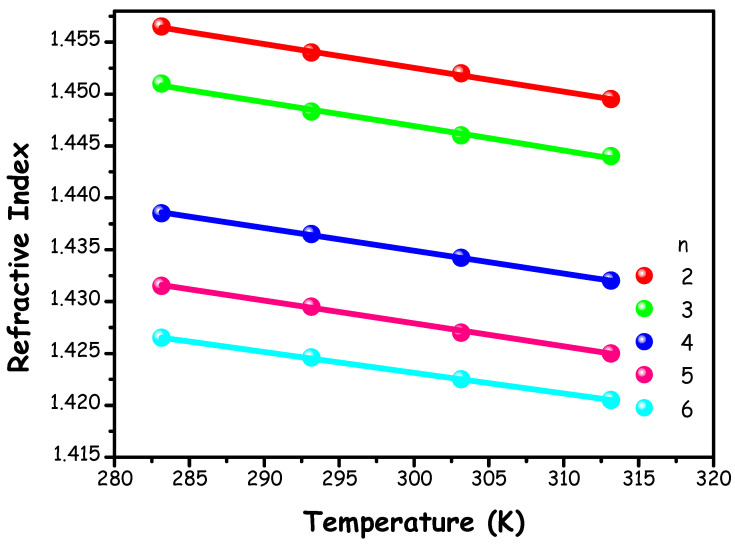
n_D_ values of ChAc/water mixtures as a function of temperature. Solid lines represent linear fits.

Refractive index (n_D_) measurements were executed on the ChAc/water mixtures (n = 2–6) every 10 K over a temperature range between 283.15 and 313.15 K. The corresponding results are shown in Figure 6 and listed in Table S11. The relationship between refractive index values and temperature was described using a linear relationship, with the corresponding fitting parameters provided in Table S12 of the ESI.

The observation of a linear decrease with increasing temperature is consistent with other ChAc-based systems [19] and the ChCl/water mixture [16].

**Figure 7:**
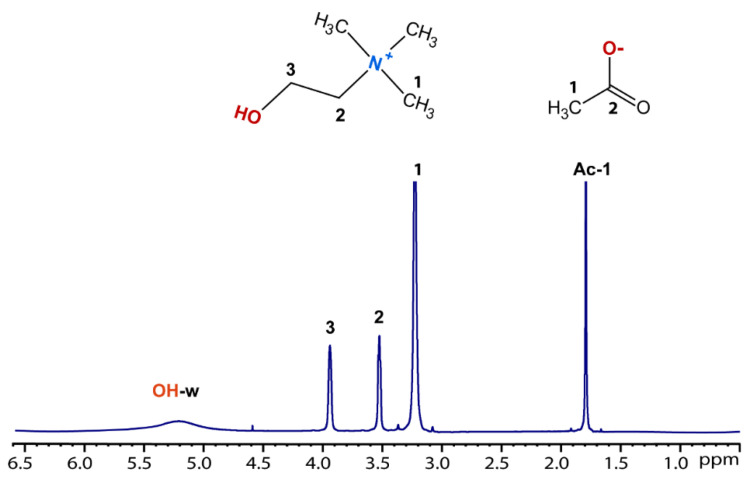
^1^H NMR spectrum of the mixture ChAc/water (n = 4), molecular structure of choline-acetate and peaks assignment.

^1^H NMR methods have been widely used to study the local structure and dynamics of several choline-based DES systems. As an example, the proton spectrum of ChAc/water (n = 4), along with the molecular structure and peak assignments, is shown in Figure 7. The spectrum shows well-resolved lines for all protons of choline and acetate, in particular we observe the coalescence of the –OH and water peaks in a broad signal indicating an intermediate dynamic exchange regime on the chemical shift timescale, analogous to our previous findings on ChCl/water [16].

The proton spectra of all samples ChAc/water (n = 2–6) are reported in Figure S5. We can observe that by increasing the amount of water, the linewidth of the –OH and water signals is progressively modified, becoming narrower. No significant changes are observed in the chemical shift of water, choline, and acetate (data shown in Figure S6 of the ESI).

Additionally, NMR diffusion studies are performed to obtain information on the translational motion of individual molecular species forming the solutions [52,53]. The diffusion coefficients of both choline and acetate (Figure S6) increase progressively with the amount of water added, with acetate faster than choline, reflecting the decrease in viscosity of the entire system. Water diffusivity maintains mainly constant for the probed ChAc/water mixtures (n = 4–6); this behavior is at odds with the one encountered in choline chloride water wb-DES, where a faster, composition dependent diffusivity was observed for water [16].

Diffusion coefficients can be described in terms of the Stokes–Einstein (SE) equation:

D = k_B_T/6π·η·R_h_ (8)

where k_B_ is the Boltzmann constant, D, η, and R_h_ are the diffusion coefficient, viscosity, and hydrodynamic radius of the probed species, respectively.

**Figure 8:**
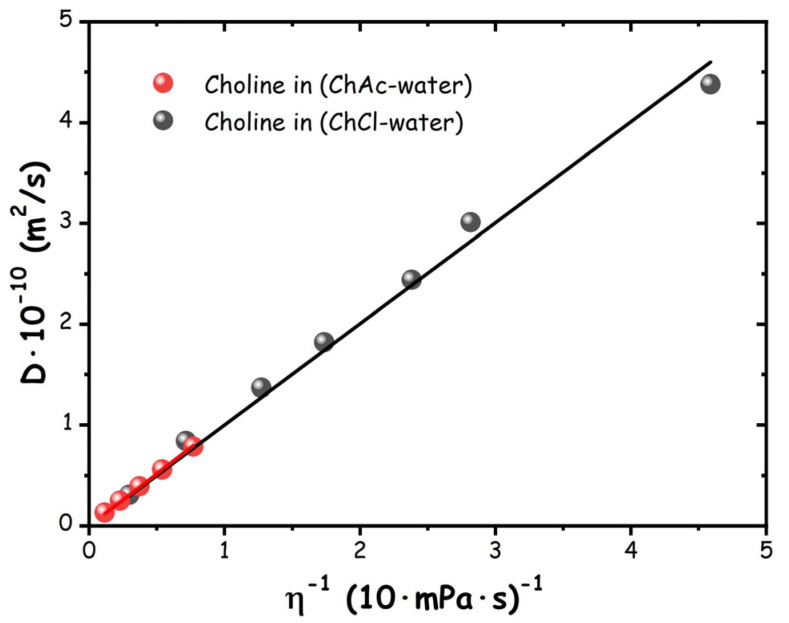
^1^H NMR diffusion coefficients of choline cation in ChCl/water and ChAc/water mixtures versus fluidity at 300 K. The straight lines correspond to a modelling of the data in terms of the SE equation with values of R_h_ = 2.19 and 2.13 Å, for the two data sets.

Figure 8 shows a comparison of diffusion coefficients of the choline cation, D_Ch_, versus the corresponding fluidities (inverse viscosities), for ChCl/water [16] and ChAc/water mixtures at 300 K, respectively. It clearly emerges that the two data sets are essentially superimposed and can be both described by R_h_ of ca. 2.15 Å, accounting for the diffusing choline cation. These results indicate that (a) in both wb-DES systems, across the whole explored water–salt ratio range, no evidence of change or evolution of R_h_ is observed; (b) the observed R_h_ for choline in the two probed systems is essentially identical. If an association (such as an aggregation) process took place in these systems, one would observe a change in the corresponding size of the aggregates with composition and with anion. We can then extend our original finding [16] and state that the experimental results for diffusion coefficients do not support the claim that “cluster aggregation” is observed in wb-DES (including the ChCl/water one) [54]. The report mentioned based this claim on the ground of conductivity, ultrasound spectroscopy, and diffusion coefficients for ChCl/water mixtures. While a discussion on the other characterizations is outside the scope of this work, the latter data set, which is directly comparable to our results, was not taking into consideration the D_Ch_ dependence on viscosity. Once explicitly accounting for viscosity, those D_Ch_ data nicely follow (data digitalized from the original work) the linear trend reported in Figure 8, thus indicating that the observed monotonical increasing in D_choline_ upon dilution, rather than reflecting a decrease in R_h_ (hence the wrong claim of large aggregates at low water content), is simply a consequence of decreasing viscosity, without substantial structural changes involved.

**Figure 9:**
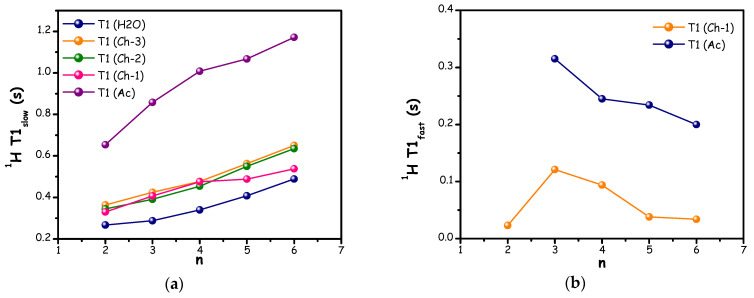
^1^H longitudinal (spin-lattice, T1) relaxation times for ChAc/water mixtures as a function of n, at 300 K T1_slow_ (**a**), T1_fast_ (**b**).

The longitudinal relaxation time (spin-lattice, T1) of all carbon and proton atoms of the mixtures ChAc/water (n = 2–6) were measured at 300 K (Figure S7 of the ESI and Figure 9).

The T1 values of all carbon atoms were obtained using a mono-exponential fit of the experimental data. In all cases, the T1 values of Ac-1 and Ac-2 are higher than those of Ch-1, Ch-2, and Ch-3 which in turn show similar values. For all carbons, the T1 increases upon water addition, indicating a fast rotational motion which can be related to a progressive decrease in viscosity of the sample.

The ^1^H T1 relaxation values (data shown in Figure 9) were best obtained by a biexponential fit of the signal decay curve, providing two relaxation components (T1_slow_ and T1_fast_) for Ch-1 and Ac protons, while Ch-2, Ch-3, and water signal decays were well fitted with a mono-exponential function. This behavior can be attributed to some dynamical heterogeneity experienced by Ch-1 and Ac in these peculiar systems.

**Scheme 1:**
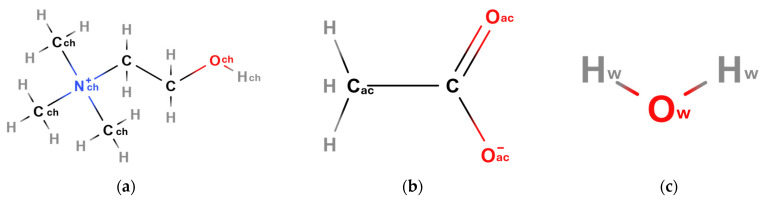
Schematic representation of choline (Ch) (**a**), acetate (Ac) (**b**), and water (**c**).

The measured T1_slow_ of all protons in choline-acetate/water mixtures (n = 2–6) increases with increasing water content (Figure 9a). In a previous study on a series of betaine-based DESs, a minimum in the T1 of the hydroxyl proton was observed and attributed to the formation of a deep eutectic characterized by a strong hydrogen-bonding network, which restricts molecular rotational motion [55]. In the present study, since no measurements were performed for n < 2, it is not possible to identify a clear minimum. However, it is evident that the lowest ^1^H T1slow value is observed at n = 2, particularly for acetate protons (Ac). This suggests a reduced mobility of acetate at this composition, which likely arises from strong hydrogen-bonding interactions involving the acetate anion (Scheme 1).

Moreover, the methyl group of acetate shows the highest T1 value. The T1 measurements were also performed at a higher temperature (320 K) and the experimental data are reported in Figures S8 and S9. A similar trend is also observed at the higher temperature, but with higher T1 values for all protons.

**Figure 10:**
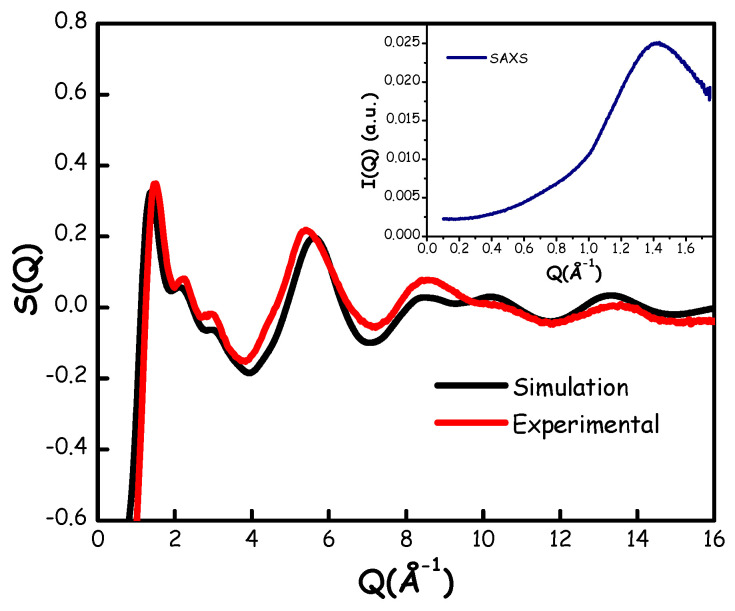
Experimental (black line, room temperature) and computed (red line, 350 K) X-ray weighted normalized structure factors for the ChAc/water mixture (n = 2). In the inset, the experimental SAXS pattern for the same mixture at room temperature.

Although the ^1^H longitudinal relaxation time (spin-lattice, T1) data do not allow us to unequivocally identify the formation of a DES, due to the absence of experimental data at n < 2, they clearly indicate the presence of a strong hydrogen-bonding network in the ChAc/water mixture at n = 2. For this reason, and considering that several known DESs are based on a 1:2 HBA:HBD molar ratio, we selected this composition for detailed structural analysis, as it offers a meaningful basis for comparison with other DES systems. Thus, a structural examination of the mixture at this composition was carried out using ab initio molecular dynamics (AIMD) simulations. The simulated density is 1.0345 g/cm^3^, to be compared with an extrapolated value from experimental data of 1.06526 g/cm^3^, with a satisfactory agreement by less than 3%. In Figure 10, a comparison between computed (at 350 K) and synchrotron X-ray weighted normalized structural factor (at 298 K) is presented. The satisfactory agreement between computed and experimental data (especially considering the difference in temperature between the two data sets) validates the AIMD simulation from the structural point of view. Experimental scattering data at low Q-values, reported in the inset of Figure 10, do not indicate evidence of structural heterogeneities in the mesoscale, similarly to what we reported in the case of both the ChCl/water [16,17] and ChAc–urea [20] systems. Together with the evidence related to diffusion coefficients discussed above, these findings further indicate that, analogously to other systems (ChCl/water and ChAc–urea), the presently studied wb-DES are not characterized by mesoscopic structural heterogeneities, related to “cluster aggregation”. On the other hand, the existence of an intense and structured feature at ca. 1.5 Å^−1^ is common in liquid systems with organic chemicals, including ChCl-based DESs [9,17,33,56,57,58,59,60].

**Figure 11:**
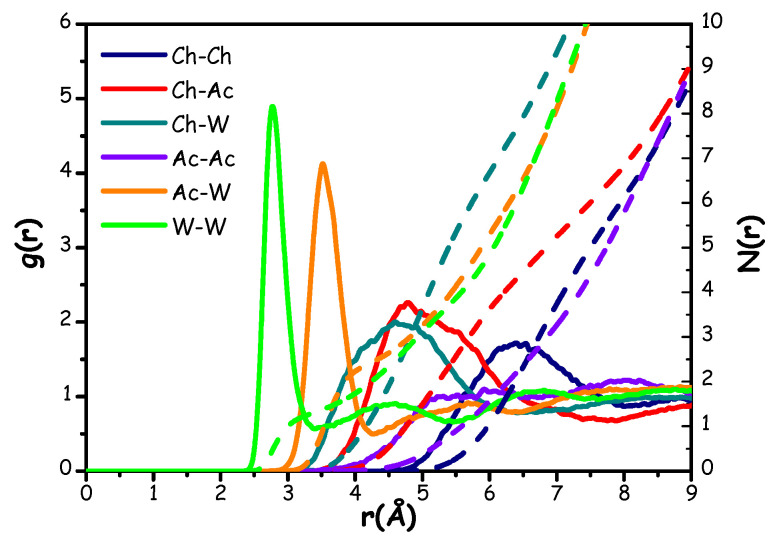
Radial distribution functions (solid lines) and related number of nearest neighbors (dashed lines) of the centers of mass of choline (Ch), acetate (Ac) and water (W).

The AIMD generated trajectory was interrogated for further structural details, by examining the radial distribution functions (RDFs) and the related running numbers of neighbors (N(r)s) of selected moieties/atomic species. In Figure 11, the RDFs relating to the correlations between the centers of mass of all the species in the mixture are shown.

Figure 11 illustrates a situation where the correlations between the centers of mass are distributed across three distinct regions, akin to the pattern observed in a previous ChCl-based wb-DES, named Aquoline (ChCl:water 1:3.3) [17]. This effect is likely due to the difference in size between the different components: (*i*) at distances r < 3 Å, a sharp and intense peak centered at ca. 2.8 Å reflects the strong correlation between neighbor water molecules (W–W correlation); (*ii*) correlations involving W and either Ac or Ch, as well as the Ch–Ac and Ac–Ac ones, fall in the intermediate range between 3.0 Å and 6.0 Å; finally, (*iii*) Ch–Ch correlations are characterized by a broad, weak peak above 6 Å (at ca. 6.4 Å).

In the intermediate range, the Ac–W correlation shows a pronounced peak at around 3.5 Å, while Ch–Ac and Ch–W correlations are characterized by broader and less intense peaks centered at 4.9 Å and 4.6 Å, respectively. This prompts strong interactions between the acetate anion and water molecules. For comparison, the Ch–Ac correlation in the ChAc–urea system shows a peak centered at a greater distance of approximately 5.3 Å, along with a lower number of acetate anions surrounding a reference choline (about 3.4 vs. the present value of ca. 6 (see Table 1) [20]. The Ch–W interaction, on the other hand, resembles that reported in Aquoline, although the peak here is centered at a slightly lower distance (4.6 Å compared to 5.0 Å [17]).

Ac–Ac correlation appears to be relatively featureless and with an amplitude close to unity, thus reflecting a low probability of interactions likely due to electrostatic repulsion between acetate anions. Indeed, we can observe only a broad peak with a very low intensity around 8.0 Å. This observation aligns with the ChAc–urea mixture [20], but contrasts sharply with the findings in ChCl/water [17], where the anion–anion correlation exhibited a more structured pattern.

The observations related to Ch–Ch correlations at large separation distances are consistent with those in Reline, Aquoline, and the ChAc–urea system [17,20,56,60], where a peak around 6.5 Å was detected. Furthermore, integrating this peak up to a distance of about 8.0 Å yields a number of nearest neighbors equal to 6.2, which resembles the cases of both Reline (ChCl–urea 1:2) [56] and Aquoline [17], while, in the case of the ChAc–urea system, only 3 surrounding choline neighbors were found [20]. In general, the observed overall spatial distribution is quite similar to the one seen in Aquoline, but with one key difference: in Aquoline, the correlation between the anion and water occurs in the first region (r < 3 Å), whereas in this case, it is observed in the central region (r between 3.0 Å and 6.0 Å) [17]. This discrepancy is likely related to the smaller size of chloride compared to acetate. Moreover, it is worth noting that the RDFs for the correlations between choline and either water or acetate nearly overlap between 4 and 6 Å. This suggests a strong interplay between the acetate anion and water in efficiently solvating the choline cation.

**Figure 12:**
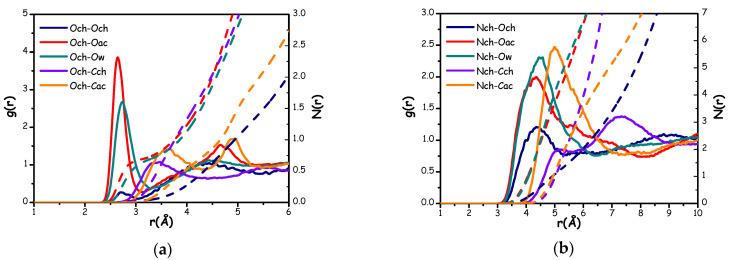
Radial distribution functions (solid lines) and corresponding number of nearest neighbors (dashed lines) for correlations between the Och group and selected atomic species of choline, acetate, and water (**a**), and between the Nch group and selected atomic species of choline, acetate, and water (**b**).

To obtain a clearer understanding of the overall structural organization, particularly around the choline cation, we further examined the RDFs associated with the correlations involving the Och or Nch atoms with selected atomic species from choline, acetate, or water (Figure 12).

Figure 12 shows that both water and acetate effectively solvate the choline oxygen region and the ammonium moiety. However, more structured features are observed in the RDFs related to the Och species. This may be attributed to the steric hindrance caused by the methyl groups in the choline ammonium moiety and to the development of hydrogen bonding interactions involving the choline hydroxyl group. Specifically, both the Och–Oac and Och–Ow correlations exhibit sharp, intense peaks centered at approximately 2.6 Å and 2.7 Å, respectively, with comparable solvation numbers 0.7 (Figure 12a). When comparing with the behavior observed in Aquoline [17], here, a larger role played by the anion is observed, but this observation is presumably a consequence of the lower water content in the present case.

On the other hand, both the Och–Cch and Och–Cac correlations are characterized by less sharp and intense peaks, centered at distances of 3.5 Å and 3.6 Å, respectively (Figure 12a). This suggests weak interactions between hydroxyl moiety and methyl groups. In the case of Cch, the observed correlation likely arises from the coulombic interactions between the positively charged ammonium group and the partially negatively charged hydroxyl moiety. In the case of Cac, the correlation is likely mediated by the interaction between the choline hydroxyl hydrogen and acetate oxygen atoms. Finally, the Och–Och correlation clearly exhibits an unstructured peak, indicating a low probability of interaction between two choline molecules.

Concerning Nch, water and acetate provide efficient solvation. Both the Nch–Ow and Nch–Oac correlations exhibit the highest intensity peaks (Figure 12b), and at the same integration distance, they show the greatest number of nearest neighbors (Table 2). Interestingly, at a relatively short distance of about 4.4 Å, a moderately intense peak is observed for the Nch–Och correlation. This correlation might be attributed to the electrostatic attraction between the positively charged ammonium group and the choline oxygen, but also to a collective solvation scenario induced by both acetate and water, with simultaneous solvation of both the ammonium group and the hydroxyl one.

Finally, examining the RDFs that involve correlations between Nch and Cac, we observe a relatively intense peak around 5.0 Å. This peak reflects the ammonium solvation scenario involving the acetate oxygens. In the case of the Nch–Cch correlation, a broad peak is observed at approximately 7.3 Å, accompanied by a small shoulder just below unity around 5.1 Å (Figure 12b). This feature suggests the difficulty that the choline methyl group faces in solvating the ammonium group of another choline molecule.

Interestingly, for both the Och and Nch species, all correlations involving acetate or water show similar features and numbers of nearest neighbors (Table 2). However, for the Och species, the acetate anion is closer to the hydroxyl group than the water molecules. A similar trend is observed for the Nch species, where the Nch–Oac correlation occurs at a shorter distance than the Nch–Ow correlation (Table 2).

**Figure 13:**
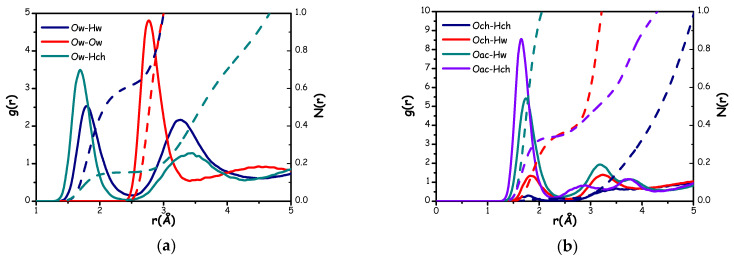
Radial distribution functions (solid lines) and corresponding number of nearest neighbors (dashed lines) for selected hydrogen bonding correlations considering water as the reference molecule (**a**) and choline or acetate as the reference molecule (**b**).

A better understanding about the structural organization of the probed mixture can be achieved considering all conceivable hydrogen bonding interactions between the components. In Figure 13, the correlations for all the hydrogen bonding interactions involving water or acetate as a reference are illustrated.

As shown in Figure 13, water molecules effectively solvate both hydroxyl choline and acetate oxygens’ region, with all hydrogen bonding correlations displaying sharp and intense peaks. Indeed, peaks are centered in a distance range between 1.7 and 1.9 Å and exceed unity, indicating strong hydrogen bonding interactions. However, a distinct preference of water hydrogens to interact with the acetate oxygens rather than the choline oxygen clearly emerges, as evidenced by the higher coordination number for the Oac–Hw correlation than for the Och–Hw one (see Table 3).

The hydrogen bonding solvation of water around the choline hydroxyl group shows subtle differences also when comparing the Ow–Hch and Och–Hw correlations (Figure 13a,b, respectively). The former exhibits a peak at a shorter distance than the latter (Table 3), and this peak also appears more intense and sharper. These features indicate a slight preference for water molecules to interact with the choline hydroxyl group via the Ow species, rather than through its Hw atoms. This behavior likely reflects the system’s tendency to maintain strong hydrogen bond interactions between the water hydrogen atoms and the acetate oxygen atoms.

A further inspection of Figure 13 reveals acetate anion’s strong ability to interact with the choline hydroxyl group. The Oac–Hch correlation displays the most intense peak, which, as shown in Table 3, is centered at the shortest distance and for which we found 0.36 choline hydroxyl hydrogens surrounding the acetate oxygens’ region. This observation suggests the formation of a strong hydrogen bond interaction between acetate and choline hydroxyl, as previously noted in the ChAc–urea mixture [20]. These findings underscore the significance of the anion, which can interact effectively with both choline and water.

However, when comparing the solvation effectiveness of water and acetate toward the choline hydroxyl group, the acetate anion appears to be more effective. The number of coordinating Oac species interacting with the choline hydroxyl hydrogen (Hch) exceeds the combined number of coordinating water species, both Ow and Hw, towards choline hydroxyl moiety (Table 3). This indicates that acetate oxygens may form stronger interactions with the choline hydroxyl group than water molecules do.

An examination of the Och–Hch correlation from Figure 13 highlights the difficulty of the choline cation in forming hydrogen bonds with another choline, further suggesting limited interactions between choline cations through hydrogen bonding. At the same time, although the Ow–Hw correlation displays a significant feature with a sharp peak centered at 1.8 Å, the overall analysis of Figure 13 suggests that water molecules struggle to exist in an environment similar to bulk water. This hypothesis is further supported by the Ow–Ow correlation, which, like in the Aquoline case, shows a depletion region within the first solvation shell [17]. This likely indicates a preferential interaction between water molecules and both choline and acetate, rather than with other water molecules.

**Figure 14:**
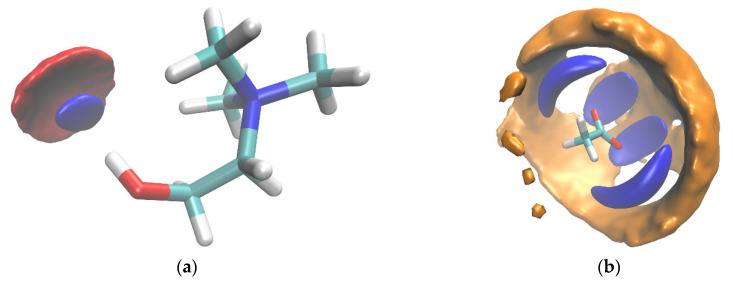
Spatial distribution function of water (blue) and acetate (red) centers of mass surrounding a reference choline hydroxyl group (**a**). Spatial distribution function of water (blue) and choline (orange) centers of mass surrounding a reference acetate anion (**b**). In both cases, the distribution isovalues refer approximately to 30% of the maximum amplitude.

In summary, the analysis of Figure 13 suggests a structural arrangement for which the role of the acetate anion, and its synergy with water, is particularly significant. The acetate, with its carboxyl group bearing a strongly delocalized negative charge, effectively interacts with both the Hch and Hw groups. Meanwhile, water molecules seem to arrange themselves around the acetate anion in such a way that they can interact with the Hch group through their Ow species, and with both the Oac and Ow of other water molecules via their Hw species. This type of structural organization can be clearly visualized through the Spatial Distribution Functions (SDFs) shown in Figure 14.

Upon examining the spatial distribution of both acetate (red) and water (blue) around the hydroxyl group of choline, it becomes clear that both species synergically interact with the hydroxyl hydrogen of the choline cation (Figure 14a). The distributions of acetate and water around the hydroxyl hydrogen nearly overlap. An inspection of Figure 14b shows how water (in blue) can distribute around acetate in a specific organized way, through which it can interact with both the oxygens’ acetate and other water molecules through strong hydrogen bonding interactions. In this way, choline cation (in orange) can effectively distribute around both acetate and water, interacting with both through its hydroxyl and ammonium region (Figure 12).

This well-defined, cooperative solvation structure, predominantly governed by strong hydrogen-bonding interactions, is characteristic of deep eutectic systems. However, due to the lack of experimental data at lower water contents, a definitive classification of the n = 2 mixture as a wb-DES cannot be firmly established.

**Figure 15:**
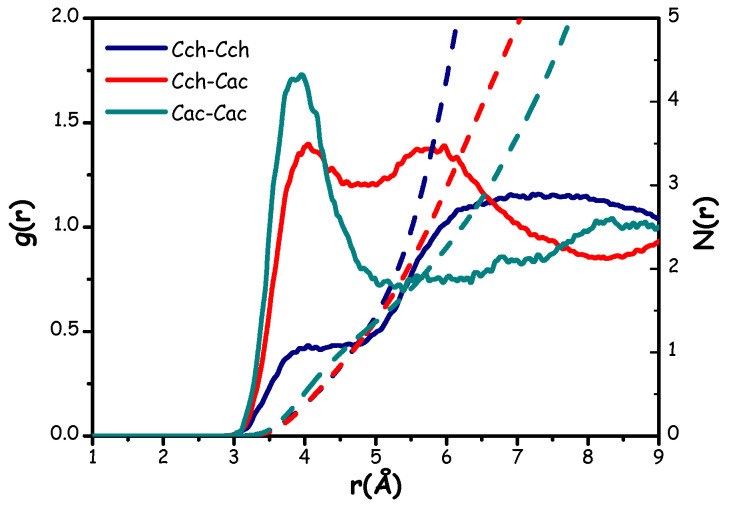
Radial distribution functions (solid lines) and corresponding number of nearest neighbors (dashed lines) for selected correlations between the methyl groups of choline and acetate.

Finally, we consider the potential for observing dispersive correlations involving the methyl groups of both choline and acetate. To illustrate this, in Figure 15, the RDFs corresponding to the correlations between the carbon atoms of the acetate and choline methyl moieties are presented. Figure 15 reveals that the correlation between two different choline methyl groups (Cch–Cch) is unstructured, showing a weak feature at 4 Å. This further prompts for a scenario where the choline cation is effectively solvated by both water and acetate, thereby reducing the likelihood of direct interactions between choline moieties.

In contrast, the Cac–Cac correlation is characterized by a sharp, intense peak centered around 3.9 Å. This feature aligns well with what was observed in the ChAc–urea mixture with n = 2, where correlations between two different acetate molecules only show significant features in the methyl region [20]. Finally, the Cch–Cac correlation exhibits a feature characterized by a broad solvation shell centered around 4.0 Å, which can be attributed to the solvation process induced by the acetate oxygens toward the positively charged ammonium group of choline. Overall, despite differences in the homogeneity of local distribution, methyl–methyl correlations seem to be driven mostly by steric hindrance, as the first solvation shells have essentially identical coordination numbers.

## 3. Discussion

In this study, a comprehensive experimental physicochemical characterization of choline acetate (ChAc)/water mixtures was performed across various water:ChAc molar ratios (n = 2–6), revealing key insights into their properties and classification. DSC analyses confirmed the absence of first-order transitions and the presence of glass transitions (T_g_) between 150 and 180 K, indicating that all systems behave as Low Transition-Temperature Mixtures (LTTMs). A comparison with thermal data reported by Miao et al. [32] suggests that their so-called “melting points” likely correspond to enthalpy recovery phenomena embedded within the glass transition, rather than true melting events. This reinforces the idea that no solid–liquid equilibria are present in these systems and that previous claims of DES behavior at n = 5 are not supported by experimental evidence.

Among all compositions, the n = 2 mixture displays distinct structural and dynamic features, as evidenced by ^1^H and ^13^C NMR relaxation data and AIMD simulations, that point to enhanced hydrogen bonding, restricted molecular mobility, and a highly structured solvation environment. These characteristics support the hypothesis that n = 2 may behave as a water-based Deep Eutectic Solvent (wb-DES), although confirmation is limited by the lack of data at lower water contents. AIMD simulations further revealed that the acetate anion plays a central role in solvating the choline cation, in synergy with water, forming a well-organized hydrogen-bond network, particularly around the hydroxyl group.

In addition, WAXS analyses, together with NMR-derived hydrodynamic radii (R_h_), showed no signs of mesoscale aggregation or heterogeneity across all mixtures, challenging recent hypotheses of clustering in similar systems [54].

Although the classification of the n = 2 mixture as a wb-DES remains tentative, the observed cooperative solvation effects and homogeneous microstructure provide compelling support in that direction. Overall, this work underscores the importance of combining thermal, spectroscopic, and computational techniques to guide the rational development of tunable, green solvent systems for applications in sustainable chemistry and environmentally friendly technologies.

## 4. Experimental Methods

### 4.1. Chemicals

**Scheme 2:**
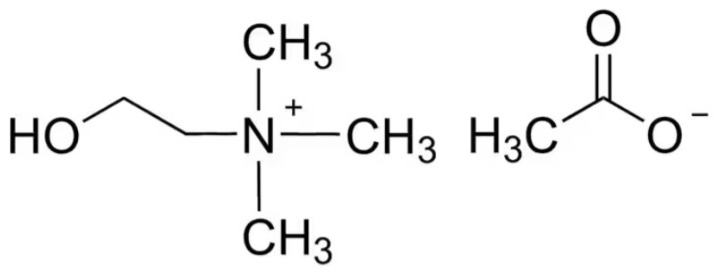
Schematic representation of ChAc.

Choline Acetate (ChAc, Scheme 2) was purchased by Iolitec (purity: 98%). Prior to the preparation of ChAc/water mixtures, ChAc was dried under high vacuum at 303 K for approximately 8 days to remove any trace of moisture and then stored under anhydrous conditions. The dried ChAc was precisely weighed inside a glove box, and MilliQ water was subsequently added to prepare mixtures with different water:ChAc molar ratios n (n = 2, 3, 4, 5, and 6, corresponding to molar fraction 0.33 > x_ChAc_ > 0.14). Mixtures were continually stirred at around 303 K until transparent and homogenous liquids were achieved.

### 4.2. DSC Measurements

DSC experiments were performed using a Mettler Toledo DSC 822 with an FRS5 sensor (Mettler-Toledo, Milan, Italy) and provided with a liquid nitrogen cooling system (STAR 10.0). During experiments, the furnace was purged with dry nitrogen at a flow rate of 30 mL/min. The samples, ranging in weight from 5 mg to 9 mg, were carefully weighed using a Mettler MT5 analytical balance (uncertainty of 0.005 mg, Mettler-Toledo, Milan, Italy). The samples were then placed in a 40 μL metal pan and quickly sealed.

Finally, mixtures were subjected to a temperature program that included dynamic cooling from 293 K to 143 K at a rate of 10 K/min, followed by heating from 143 K to 319 K with a scanning rate of 10 K/min.

### 4.3. Density Measurements

Density (ρ) experiments were conducted using a Mettler Toledo Density Meter DM45 DeltaRange (Mettler-Toledo, Milan, Italy) at temperatures ranging from 278.15 K to 323.15 K. A standard calibration procedure using dry air and bi-distilled water at 293.15 K was conducted.

### 4.4. Viscosity Measurements

An Anton Paar micro viscosimeter (Anton Paar, Graz, Austria), the Lovis 2000 M/ME model, was used to measure dynamic viscosity (η) with a 0.5% accuracy. Peltier elements were used to regulate the temperature to within 0.02 K. Calibrated glass capillaries with varied sizes (1.59 mm, 1.80 mm, and 2.50 mm) were then used for measurements and chosen depending on the desired viscosity range. These measurements took place within a temperature range of 278.15 K to 323.15 K.

### 4.5. Electrical Conductivity Measurements

A Mettler Toledo Five Easy FE30 conductivity meter (Mettler-Toledo, Milan, Italy) was used to measure electrical conductivity (ec) at temperatures ranging from 278.15 K to 313.15 K. The instrument used an LE703 conductivity electrode (Mettler-Toledo, Milan, Italy) with a cell constant of 0.73 cm^−1^. Temperature was controlled using an adequate experimental setup that included a thermostatic bath, with an accuracy of 0.1 K.

### 4.6. Refractive Index Measurements

The refractive index (n_D_) values at the Na D line (589 nm) were determined using an Abbe Refractometer 2AWJ from Optika (Ponteranica, Italy). The instrument can measure refractive indices between 1.3 and 1.7 with a resolution of 0.0005. The refractometer is equipped with a thermostat covering the temperature range of 278.15 K to 343.15 K. Measurements were carried out at intervals of 10 K within the temperature range from 283.15 K to 313.15 K.

### 4.7. X-Ray Scattering Experiments

#### 4.7.1. Small Angle X-Ray Scattering

Small-angle X-ray scattering (SAXS) experiments were carried out at the SAXSLab, Sapienza University of Rome, using a Xeuss 2.0 Q-Xoom system (Xenocs SA, Sassenage, France). The instrument is equipped with a micro-focused Genix 3D X-ray source (λ = 0.1542 nm) and a two-dimensional Pilatus3 R 300K detector, which allows for variable sample-to-detector distances. The scattering vector Q, defined as Q = (4πsinθ)/λ, with θ representing the scattering angle, was calibrated using a silver behenate reference standard. To cover a broad Q-range (0.1 < Q (Å^−1^) < 1.7), measurements were performed at multiple sample-detector distances. The probed sample was sealed in a quartz capillary (1.0 mm nominal thickness) using hot glue and placed in the instrument’s sample chamber under reduced pressure (~0.2 mbar). The raw two-dimensional scattering images were corrected for dark current, masked, azimuthally averaged, and normalized with respect to incident beam intensity, exposure time, and solid angle per pixel. All data reduction steps were carried out using the FoxTrot software (8.5) developed at SOLEIL. Subsequently, the capillary background was subtracted to obtain the final one-dimensional scattering profiles (I(Q) vs. Q).

#### 4.7.2. Wide Angle X-Ray Scattering

The X-ray scattering experiment was performed at Diamond Light Source (UK) using the I15-1 XPDF line with an energy of 76.69 keV (0.161669 Å). Data were acquired on a Perkin Elmer XRD 4343CT (Perkin Emer, Shelton, CT, USA) positioned at a distance of 700 mm, with a beam stop put on the detector to gather low angle scattering. The sample was put into a 2 mm borosilicate capillary and properly sealed with hot glue. Capillary was then put onto the beamline using an automatic robotic system and the measurements was executed for approximately 10 min at room temperature, with relevant background and empty capillary readings collected for processing.

Acquired data were then processed into 1D data using DAWN (2.38) [61]. Corrections were applied for background, absorption, polarization and Compton scattering using GudrunX (2017) [62].

### 4.8. NMR Measurements

All samples were transferred to 5 mm NMR tubes, equipped with a capillary containing deuterated water (D_2_O), used as chemical shift reference and for lock. NMR measurements were performed on a Bruker NEO 500 console (11.74 T, Bruker, Milan, Italy) equipped with a direct observe BBFO (broadband including fluorine) iProbe. ^1^H spectra were recorded with 4 scans using 32,768 points and SW = 8 ppm. ^1^H and ^13^C-{^1^H} T1 relaxation measurements were carried out using standard inversion recovery (IR) technique. In this case, the temperature was set at 300–320 K and controlled with air flow. ^13^C-{^1^H} spectra were acquired for delay time τ, in the range 0.05–200 s and data matrices of 16,384 (t2) × 16 (t1), with 8 transients per increment, over a spectral width of 200 ppm. ^1^H data were recorded for various delay time τ, in the range 0.05–8 s with and data matrices of 16,384 (t2) × 16 (t1), with 8 transients per increment, over a spectral width of 8 ppm. Data were processed using a mono-exponential or bi-exponential fitting function (R^2^ = 0.999). ^1^H diffusion coefficients were measured using the bipolar pulse longitudinal eddy current delay (BPP-LED) pulse sequence. The pulse gradients were incremented from 2 to 95% of the maximum gradient strength in a linear ramp with 32 steps using a pulsed gradient unit capable of producing sine-shaped magnetic field pulse gradients in the z-direction of 53 G cm^−1^. All experiments were carried out with pulse gradient δ = 2.4–3 ms, diffusion time Δ = 0.2–0.6 ms, 8 scans, and a relaxation delay of 10 s. Experimental errors are within 2%.

### 4.9. Computational Details

To investigate the n = 2 system by means of quantum chemical methods, ab initio molecular dynamics (AIMD) simulations have been performed with the CP2k program package (2025.2) [63,64], using the Quickstep method [65] and Orbital Transformation (OT) [66]. The electron structure was described by Kohn–Sham density functional theory (KS-DFT) [67,68] with the BLYP exchange-correlation functional [69,70] together with the empirical D3 dispersion correction [71,72], using the revised damping parameters of Smith et al. [73]. Atom-centered basis sets of the type DZVP-MOLOPT-SR-GTH [74] and GTH pseudopotentials [75,76] were applied, and the plane-wave energy cutoff was set to 350 Ry. The simulation was performed in NVT ensemble; the temperature was kept at 350 K with a Nosé–Hoover chain (NHC) thermostat [77,78] using a time constant of 100 fs. The equations of motion were integrated with time step of 0.5 fs. The AIMD simulation was running for 330 ps (660,000 steps), of which the first 50 ps were discarded as equilibration.

The initial configuration for the AIMD simulation was obtained as follows: 35 ion pairs of ChAc and 70 water molecules were randomly distributed in a cell using the Packmol software (v21.0.4) [79]. An initial pre-equilibration was performed via force field MD using the Lammps package [80], modeling water with the TIP4P-EW force field [81] and ChAc with Acevedo’s parameters [82]. The bonds and angles in water were kept fixed with the SHAKE algorithm [83,84]. The Lennard-Jones and Coulomb cutoff was set to 800 pm, and long-range electrostatic interactions were described by a PPPM solver as implemented in Lammps. The simulation protocol was as follows. After a short (25 ps) NVT simulation with a Berendsen thermostat [85] (coupling constant 5.0 fs) at 450 K, a first NpT run for 1.0 ns followed, using a Nosé–Hoover thermostat [77,78] (coupling constant of 100 fs) and a Nosé–Hoover barostat (coupling constant of 2000 fs). Subsequently, the temperature was ramped down to 350 K in NVT ensemble, followed by another NpT run for 500 ps (parameters as above). Then, a Langevin thermostat [86,87] was applied for 500 ps to dampen acoustic shock waves in the system due to the changing cell size. During a long (4 ns) NpT run, the average cell density was computed, and the cell size was then scaled to this value and kept fixed from now on. The temperature was again ramped up to 450 K to allow for the proper orientation of the molecules, and after additional 2 ns simulation at increased temperature, the system was cooled down to 350 K. Finally, 20 ns of equilibration were performed in NVT ensemble to obtain the initial configuration for the AIMD simulation. The resulting cell density was found to be 1.035 g cm^−1^, corresponding to a cubic cell size of around 2.2 nm.

The radial distribution functions (RDFs) and spatial distribution functions (SDFs) were computed from the AIMD trajectory using the TRAVIS program package [88,89,90].

## Figures and Tables

**Table 1:** Peak position, distance of integration and number of nearest neighbors related to all the correlations shown in Figure 11.

Correlation	Peak Position (Å)	Integration Distance (Å)	N(r)
Ch–Ch	6.4	8.0	6.2
Ch–Ac	4.9	7.0	5.3
Ch–W	4.6	6.3	7.5
W–Ch	4.6	6.3	3.7
Ac–Ac	8.0	9.0	9.1
Ac–W	3.5	4.3	2.5
W–Ac	3.5	4.3	1.2
W–W	2.8	3.5	1.4

**Table 2:** Peak position, distance of integration, and number of nearest neighbors related to all the correlations shown in Figure 12.

Correlation	Peak Position (Å)	Integration Distance (Å)	N(r)
Och–Oac	2.6	3.5	0.83
Och–Ow	2.7	3.5	0.77
Och–Cch	3.4	4.3	1.70
Och–Cac	3.6	4.3	0.74
Nch–Och	4.4	5.5	0.63
Nch–Oac	4.3	5.5	2.20
Nch–Ow	4.5	5.5	2.30
Nch–Cch	5.1	5.5	1.03
Nch–Cac	5.0	7.5	2.50

**Table 3:** Peak position, distance of integration, and number of nearest neighbors related to all the correlations shown in Figure 13.

Correlation	Peak Position (Å)	Integration Distance (Å)	N(r)
Ow–Hw	1.8	2.5	0.62
Ow–Ow	2.8	3.5	1.4
Ow–Hch	1.7	2.5	0.16
Hw–Och	1.9	2.5	0.10
Hch–Ow	1.7	2.5	0.31
Och–Hw	1.9	2.5	0.37
Oac–Hw	1.7	2.5	1.16
Oac–Hch	1.7	2.5	0.36
Hw–Oac	1.7	2.5	0.58
Hch–Oac	1.7	2.5	0.71
Och–Hch	1.8	2.5	0.02

## Data Availability

The original contributions presented in this study are included in the article/Supplementary Materials. Further inquiries can be directed to the corresponding authors.

## Supplementary Materials

The following supporting information can be downloaded at: https://www.mdpi.com/article/10.3390/molecules30163403/s1.
