# Supplementary material for: Choline Acetate/Water Mixtures: Physicochemical Properties and Structural Organization
Source: Molecules. 2025 Aug 18;30(16):3403. doi: 10.3390/molecules30163403 (PMC12388187; doi:10.3390/molecules30163403)
Supplement: Supplementary file 1 [file molecules-30-03403-s001.zip › molecules-3797657-supplementary.pdf]

# Choline Acetate/Water mixtures: physicochemical properties and structural organization

Emanuela Mangiacapre [1], Zina Barhoumi [2], Martin Brehm [3], Franca Castiglione [4], Valerio Di Lisio [5], Alessandro Triolo [6,*], Olga Russina [1,*]

[1] *Chemistry Department, University of Rome La Sapienza, Rome 00185, Italy*

[2] *Department of Chemistry, University of Tunis El Manar, Tunisia*

[3] *Chemistry Department, Paderborn University, Paderborn, Warburger Strasse 100, 33098 Paderborn, Germany*

[4] *Department of Chemistry, Materials and Chemical Engineering "Giulio Natta", Politecnico di Milano, 20133 Milan, Italy*

[5] *Donostia International Physics Center, Paseo Manuel de Lardizabal 4, 20018 San Sebastián, Spain*

[6] *Istituto Struttura della Materia – Consiglio Nazionale delle Ricerche (ISM-CNR), Rome 00133, Italy*

| n | 2 | 3 | 4 | 6 |
|---|---|---|---|---|
| $T_g$ (K) | 173.15 | 168.15 | 163.15 | 155.15 |

**Table S1.** Glass transitions ($T_g$) for each ChAc/water mixture (n=2, 3, 4, 6).

| wt% of water | n | $T_g$ (K) | $T_m$ (K) |
|---|---|---|---|
| 0 | | | 338 |
| 18 | 2 | 173 | |
| 25 | 3 | 168 | 177[a] |
| 30 | 4 | 163 | |
| 35 | 5 | | 167[a] |
| 40 | 6 | 155 | |
| 50 | 9 | | 235[a] |
| 100 | | | |

**Table S2**. Glass transition ($T_g$) and melting ($T_m$) temperatures for each ChAc/water mixture. [a] Data obtained by Miao et al. [32].

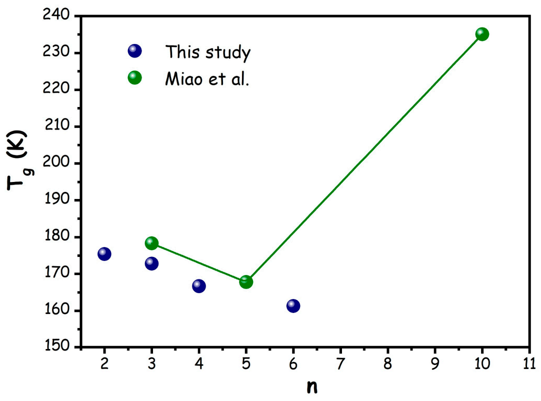


**Figure S1.** Glass transitions, $T_g$, and temperature of the transition reported by Miao et al. [1], $T_{tr}$, for ChAc-water mixtures at various values for n.

| T (K) | n=2 | n=3 | n=4 | n=5 | n=6 |
|---|---|---|---|---|---|
| 278.15 | 1.10497 | 1.09941 | 1.09529 | 1.08981 | 1.08394 |
| 283.15 | 1.10234 | 1.09679 | 1.09271 | 1.0873 | 1.08153 |
| 288.15 | 1.09956 | 1.09406 | 1.09004 | 1.0847 | 1.07901 |
| 293.15 | 1.09676 | 1.09132 | 1.08734 | 1.08206 | 1.07644 |
| 298.15 | 1.09401 | 1.08862 | 1.08468 | 1.07944 | 1.07389 |
| 303.15 | 1.09125 | 1.08588 | 1.08198 | 1.07677 | 1.07129 |
| 308.15 | 1.08848 | 1.08316 | 1.07926 | 1.0741 | 1.06867 |
| 313.15 | 1.08574 | 1.08044 | 1.07656 | 1.07142 | 1.06604 |
| 318.15 | 1.08298 | 1.07768 | 1.07381 | 1.06869 | 1.06336 |
| 323.15 | 1.08021 | 1.07491 | 1.07105 | 1.06593 | 1.06063 |

**Table S3.** Experimental density (g/cm$^3$) values for the ChAc/water mixtures at each n, as a function of temperature in a range of 278.15 K-323.15 K.

| n | 2 | 3 | 4 | 5 | 6 |
|---|---|---|---|---|---|
| **A (g·cm$^{-3}$·K$^{-2}$)** | -5.4E-08 | -7.9E-08 | -3.97E-07 | -4.51E-07 | -6.01E-07 |
| **B (g·cm$^{-3}$ ·K$^{-1}$)** | -5.20E-04 | -4.98 E-04 | -3.01E-04 | -2.60E-04 | -1.60E-04 |
| **$\rho_o$ (g/cm$^3$)** | 1.25382 | 1.24398 | 1.20958 | 1.19703 | 1.17507 |

**Table S4**. Fitting parameters of the experimental density for the ChAc/water mixtures at each n as a function of temperature, in terms of the model: $\rho(T)=AT^2 + BT + \rho_o$.

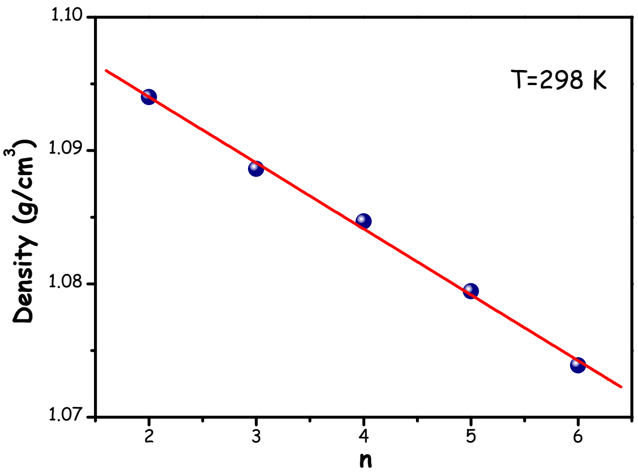


**Figure S2**. Density as a function of n for the ChAc/water mixtures at 298.15 K. The solid red line represents a linear fitting curve.

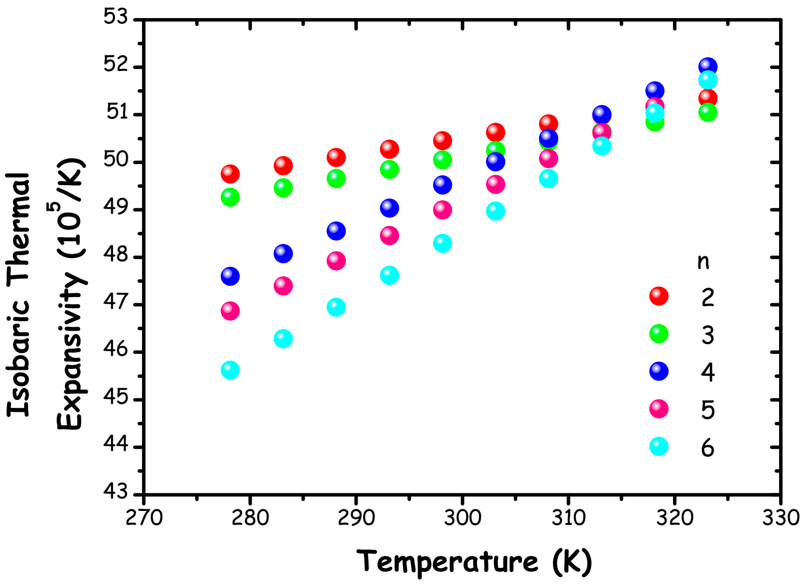


**Figure S3.** Isobaric thermal expansivity (α) values of ChAc/water mixtures at different molar ratios n as function of temperature.

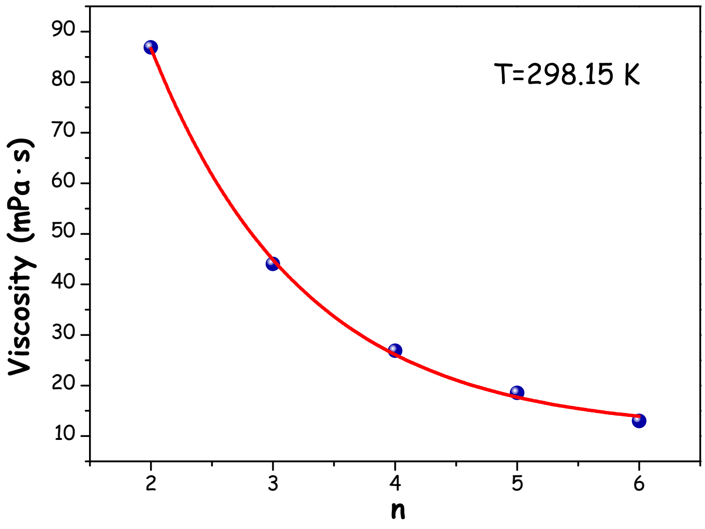


**Figure S4.** Dynamic viscosity of ChAc/water mixtures as a function of molar ratios n at 298.15 K.

| T(K) | n=2 | n=3 | n=4 | n=5 | n=6 |
|---|---|---|---|---|---|
| 278.15 | | 139.25 | 76.36 | 46.51 | 30.6 |
| 283.15 | 215.1 | 100.19 | 56.55 | 35.76 | 23.94 |
| 288.15 | 153.9 | 75.93 | 43.07 | 27.98 | 19.47 |
| 293.15 | 114.3 | 56.09 | 33.75 | 22.23 | 15.82 |
| 298.15 | 86.85 | 44.05 | 26.85 | 18.53 | 12.99 |
| 303.15 | 67.12 | 34.69 | 21.66 | 15.09 | 10.77 |
| 308.15 | 52.27 | 27.86 | 18.24 | 12.4 | 9.025 |
| 313.15 | 41.91 | 22.44 | 15.04 | 10.33 | 7.637 |
| 318.15 | 34.04 | 19.21 | 12.49 | 8.706 | 6.546 |
| 323.15 | 28.84 | 15.96 | 10.49 | 7.387 | |

**Table S5**. Experimental dynamic viscosity (mPa·s) values for the ChAc/water mixtures at each n, as a function of temperature in a range of 278.15 K-323.15 K.

| n | 2 | 3 | 4 | 5 | 6 |
|---|---|---|---|---|---|
| **$\ln\eta_0$** | -2.519 | -2.580 | -2.575 | -2.578 | -2.657 |
| **D** | 908.9 | 831.1 | 777.9 | 742.9 | 732.5 |
| **$T_0$ (K)** | 167.9 | 167.6 | 165.5 | 162.4 | 157.7 |

**Table S6.** Fitting parameters of the experimental dynamic viscosity for the ChA/water mixtures at each n as a function of temperature, in terms of the Vogel-Fulcher-Tammann model.

| T (K) | n=2 | n=3 | n=4 | n=5 | n=6 |
|---|---|---|---|---|---|
| 278.15 | 1.425 | 2.64 | 3.51 | 5.22 | 7.16 |
| 283.15 | 1.963 | 3.55 | 4.55 | 6.68 | 9.01 |
| 288.15 | 2.6 | 4.66 | 5.83 | 8.59 | 11.17 |
| 293.15 | 3.39 | 5.94 | 7.41 | 10.54 | 13.84 |
| 298.15 | 4.34 | 7.45 | 9.25 | 12.87 | 16.82 |
| 303.15 | 5.57 | 9.24 | 11.33 | 15.38 | 19.95 |
| 308.15 | 6.9 | 11.26 | 13.67 | 18.36 | 23.6 |
| 313.15 | 8.18 | 13.27 | 15.87 | 21 | 27.1 |

**Table S7.** Experimental conductivity (mS/cm) values for the ChAc/water mixtures at each n, as a function of temperature between 278.15 and 313.15 K.

| n | 2 | 3 | 4 | 5 | 6 |
|---|---|---|---|---|---|
| **$\ln k_0$** | 7.051 | 7.095 | 7.073 | 7.054 | 7.084 |
| **D** | -655.3 | -595.2 | -572.0 | -541.4 | -502.3 |
| **$T_0$ (K)** | 180.5 | 181.0 | 180.2 | 177.9 | 180.3 |

**Table S8.** Fitting parameters of the experimental electrical conductivity for the ChAc/water mixtures at each n as a function of temperature, in terms of the Vogel-Fulcher-Tammann model.

| **Λ·η (S·mPa·s/cm²·mol)** | | | | | |
|---|---|---|---|---|---|
| **T (K)** | **n=2** | **n=3** | **n=4** | **n=5** | **n=6** |
| 278.15 | | 47.85 | 57.48 | 56.22 | 54.71 |
| 283.15 | 76.23 | 46.81 | 55.31 | 55.45 | 53.98 |
| 288.15 | 72.42 | 47.16 | 54.11 | 55.92 | 54.56 |
| 293.15 | 70.31 | 44.97 | 54.02 | 54.65 | 55.06 |
| 298.15 | 68.56 | 44.54 | 53.78 | 55.76 | 55.07 |
| 303.15 | 68.18 | 44.88 | 53.27 | 54.40 | 54.29 |
| 308.15 | 65.94 | 44.01 | 54.26 | 53.49 | 53.95 |
| 313.15 | 62.84 | 41.98 | 52.07 | 51.10 | 52.55 |

**Table S9.** Λ·η values extracted from the Walden rule for the ChAc/water mixtures at each n, as a function of temperature between 278.15 and 313.15 K.

| **n** | **2** | **3** | **4** | **5** | **6** |
|---|---|---|---|---|---|
| **α** | 0.895 | 0.899 | 0.956 | 0.950 | 0.983 |
| **const** | -0.156 | -0.150 | -0.243 | -0.229 | -0.251 |

**Table S10.** Values of α and const derived from linear regression of the Walden plot for each ChAc/water mixture.

| **T (K)** | **n=2** | **n=3** | **n=4** | **n=5** | **n=6** |
|---|---|---|---|---|---|
| 283.15 | 1.4565 | 1.451 | 1.4385 | 1.4315 | 1.4265 |
| 293.15 | 1.454 | 1.4483 | 1.4365 | 1.4295 | 1.4246 |
| 303.15 | 1.452 | 1.446 | 1.4342 | 1.427 | 1.4225 |
| 313.15 | 1.4495 | 1.444 | 1.432 | 1.425 | 1.4205 |

**Table S11.** Experimental refractive index values for the ChAc/water mixtures at each n, as a function of temperature in a range of 283.15 K-313.15 K.

| n | 2 | 3 | 4 | 5 | 6 |
|---|---|---|---|---|---|
| **A** | -0.00023 | -0.00023 | -0.00022 | -0.00022 | -0.00020 |
| **B** | 1.5216 | 1.5168 | 1.5003 | 1.4938 | 1.4835 |

**Table S12.** Fitting parameters of the experimental refractive index values for the ChAc/water mixtures at each n as a function of temperature, in terms of linear trend.

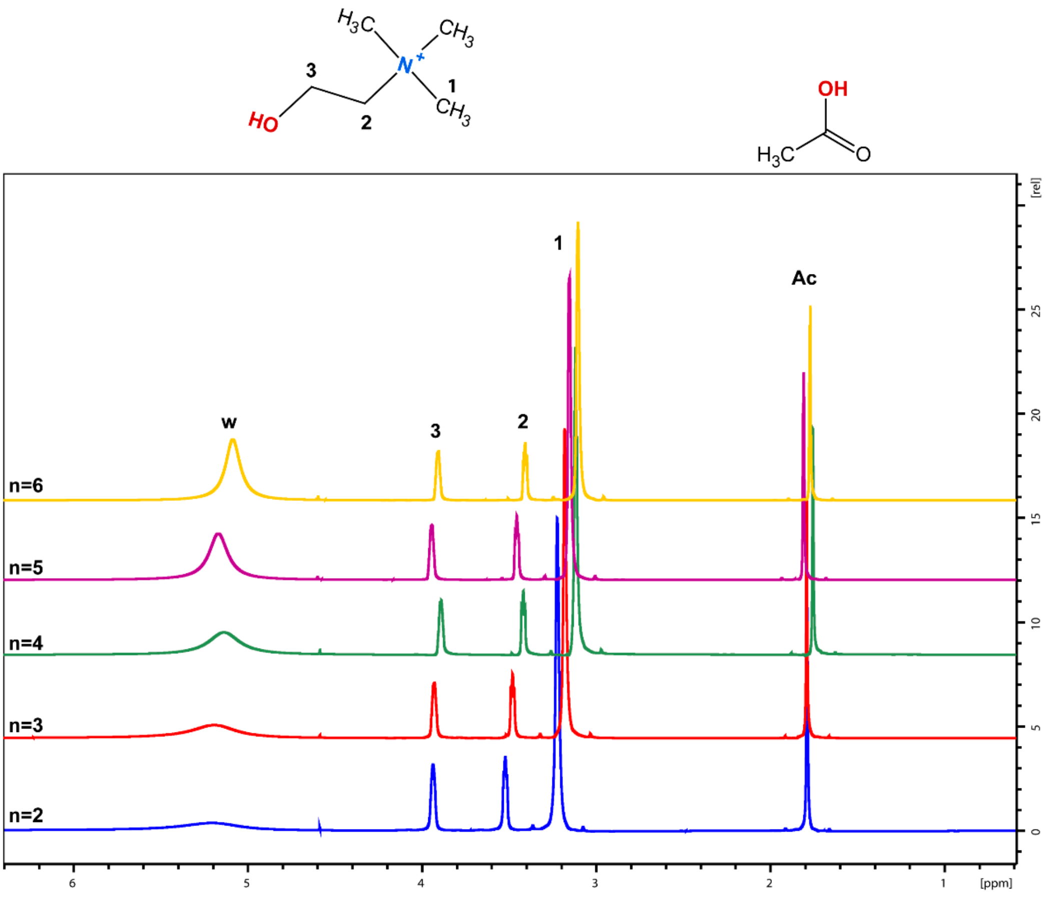


**Figure S5** $^{1}$H NMR spectra of ChAc/water mixtures at different molar ratios n, molecular structures of choline-acetate and peaks assignment.

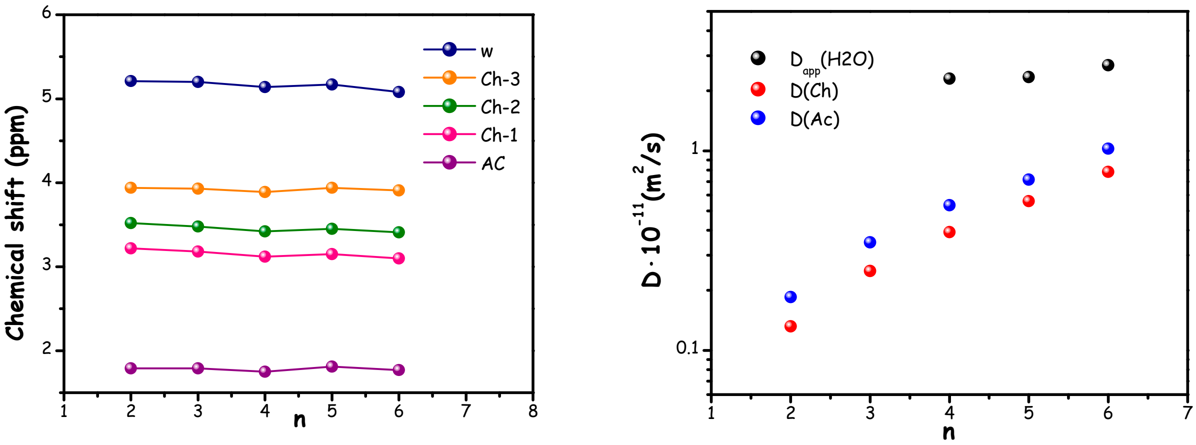


(a) (b)

**Figure S6.**[1]H chemical shifts of the different moieties in ChAC/water as a function of n (a). Diffusion coefficients of choline (Ch) and acetate (Ac), and apparent diffusion coefficient of water, in ChAc/water samples as a function of n (b). Data measured at 300 K.

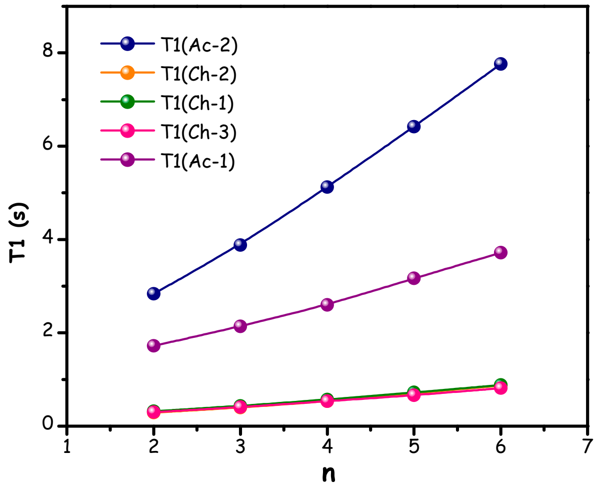


**Figure S7.** [13]C longitudinal (spin−lattice, $T_1$) relaxation times for ChAc/water mixtures as a function of n, at 300 K.

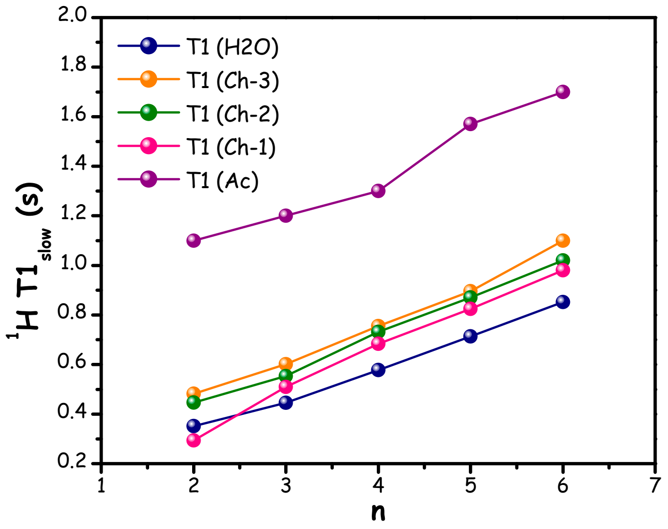


**Figure S8.** $^1$H longitudinal (spin−lattice, $T1_{slow}$) relaxation times for ChAc/water mixtures as a function of n, at 320 K.

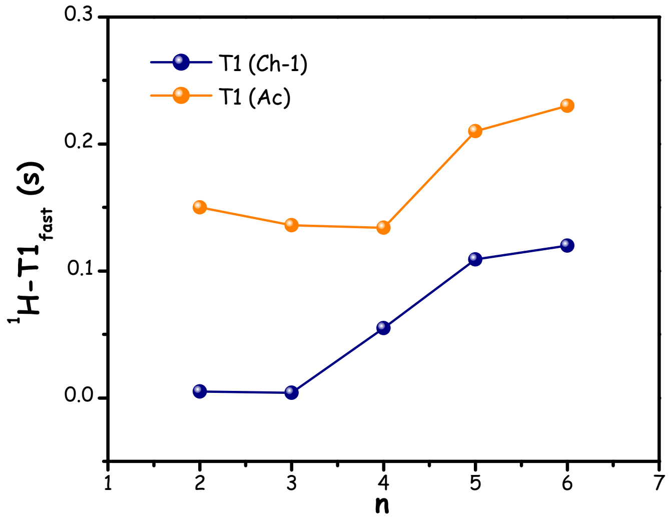


**Figure S9.** $^1$H longitudinal (spin−lattice, $T1_{fast}$) relaxation times for ChAc/water mixtures as a function of n, at 320 K.
